# Rare variants in genes coding for components of the terminal pathway of the complement system in preeclampsia

**DOI:** 10.21203/rs.3.rs-4121735/v1

**Published:** 2024-04-02

**Authors:** A Lokki, Michael Triebwasser, Emma Daly, FINNPEC COHORT, Mrtja Kurki, Markus Perola, Kirsi Auro, Jane Salmon, Anuja Java, Mark Daly, John Atkinson, Hannele Laivuori, Seppo Meri

**Affiliations:** University of Helsinki

## Abstract

Preeclampsia is a common multifactorial disease of pregnancy. Dysregulation of the complement activation is among emerging candidates responsible for disease pathogenesis. In a targeted exomic sequencing study we identified 14 variants within nine genes coding for components of the membrane attack complex (MAC, C5b-9) that are associated with preeclampsia. We found two rare missense variants in the *C5* gene that predispose to preeclampsia (rs200674959: I1296V, OR (CI95) = 24.13 (1.25–467.43), p-value = 0.01 and rs147430470: I330T, OR (CI95) = 22.75 (1.17–440.78), p-value = 0.01). In addition, one predisposing rare variant and one protective rare variant were discovered in *C6* (rs41271067: D396G, OR (CI95) = 2.93 (1.18–7.10), p-value = 0.01 and rs114609505: T190I, 0.02 OR (CI95) = 0.47 (0.22–0.92), p-value = 0.02). The results suggest that variants in terminal complement pathway predispose to preeclampsia.

## Introduction

Preeclampsia (PE) is a common pregnancy-specific vascular disorder that affects approximately 3% of pregnancies (1, 2). It accounts for over 50 000 maternal and 900 000 perinatal deaths annually (3, 4). The clinical characteristics are diverse and the course of the disease is unpredictable. The cornerstones of management are observation and delivery. Even when high quality antenatal healthcare is available, maternal morbidity is considerable and indicated preterm deliveries may result in newborn complications. There is a familial predisposition to PE and strong epidemiological evidence points to PE being partially inherited (5, 6). Furthermore, loci harbouring genes that have inflammatory or immune regulatory function were recently found to link PE and hypertension in pregnancy (7, 8).

Complement system is at the frontline of innate immunity with a capacity to cause cell death and tissue destruction as well as to trigger adaptive immune responses. In particular, the complement system has a unique capacity to discriminate between self and non-self structures and to recognize nonviable cells (9). Inadequate regulation may result in poor placentation and predispose to the development of PE (10–12). Activation of any of the three pathways of the complement system, the classical (CP), lectin (LP) or alternative pathway (AP), can lead to a common end point: terminal pathway activation featuring a membrane attack complex (MAC, C5b-9) formation. Insertion of the MAC in the plasma membrane is initiated by the C5b-8 complex composed of the C5b, C6, C7 and C8 (13). After cleavage of C5, the generated soluble C5b binds C6, which then recruits C7 and C5b67 can attach to a membrane. Further binding of C8 leads to insertion of the C5b-8 complex into a membrane. This step enables recruitment of multiple C9 molecules to complete the cylindrical MAC structure (14). In the MAC pore, C9 proteins constitute a polymeric ring with an inner diameter of about 10 nm (15). Because of a risk of membrane damage during complement activation, human cells are protected from damage by protectin (CD59). This GPI anchored protein inhibits MAC formation by binding to C5b-8 and C5b-9 to prevent further C9 from attaching to these complexes (16, 17). Therefore, the balanced regulatory capacity of CD59 and MAC activation is crucial for integrity and function of endothelial surfaces and placental trophoblast cells (CD59 is highly expressed in both of these cell types) (18).

The major activators of the CP are antibody-antigen complexes and C-reactive protein (CRP). Subsequent proteolytic activation steps by C1 lead to cleavage of C4 and C2 and formation of the CP C3 convertase (C4b2a). This bimolecular enzymatic complex is similar to the AP C3 convertase (C3bBb) and converts C3 into C3a and C3b. Contained within the AP is an efficient feedback or amplification loop for the generation of large amounts of C3b to opsonize a pathogen. Due to the feedback loop, AP activation accounts for most of the activity of the complement system (~ 80%) even if CP or LP was initially activated. Because the complement system provides a rapidly activated and potent surveillance mechanism for the host, strict control is required to avoid damage to self. Thus, inhibition of complement activation is mediated by host regulators in plasma as well as on cells. Control is aimed at each of the major steps in the pathway: initiation, amplification (leading to C3 and C5 cleavage), and formation of the MAC or C5b-9. The membrane-associated regulators are membrane cofactor protein (MCP; CD46), decay-accelerating factor (DAF; CD55) and complement receptor 1 (CR1; CD35). The fluid-phase regulators are factor H (FH) and C4b-binding protein (C4BP). During pregnancy, complement assists in the clearance of placental fragments that enter the maternal circulation as a result of syncytiotrophoblast turn-over (19). One prevailing hypothesis is that improper clearance of such components, driven by an inadequately regulated complement cascade, may lead to deposition of debris in tissues and vascular walls leading to an overly exuberant inflammatory response (11). Such unwarranted complement activation at the maternal-fetal border may lead to inadequate spiral artery remodelling, lack of maternal-fetal tolerance and poor placentation, all of which are potential key mechanisms in the pathogenesis of PE (12, 20). Genetic variants resulting in aberrant protein function in complement receptors CR3 and CR4 have been linked to increased risk of PE (19). We have recently identified and published rare genetic variants in Factor H that predispose to PE, due to excessive complement activation (21).

We designed a targeted exome sequencing protocol to screen the exomes and splicing areas of selected genes within the complement system in PE patients and controls.

## Materials and Methods

In this targeted exomic sequencing study, we combined data from exomic sequencing of 487 (body mass index < 30 kg/m^2^) PE mothers and 187 lean non-PE control mothers from the Finnish Genetics of Pre-eclampsia Consortium (FINNPEC)(22) and 122 women with a history of PE. The diagnosis of PE was based on a clinician’s diagnosis obtained through the comprehensive national Hospital Discharge Registry. Controls included 1905 parous women with no such history from the national FINRISK study (THL Biobank permit no BB2016_8). From the FINNPEC cohort, nulliparous or multiparous women with a singleton pregnancy and no history of chronic hypertension, diabetes or renal disease were included. All patients were of European ancestry. A jury consisting of a midwife and an obstetrician independently confirmed the diagnosis of PE. For the diagnosis, newly-onset hypertension and proteinuria after 20 weeks of gestation were required. Hypertension was defined as a systolic blood pressure of ≥ 140 mm Hg and/or a diastolic blood pressure of ≥ 90 mm Hg after 20 weeks of gestation. Proteinuria was defined as the urinary excretion of ≥ 0.3 g protein in a 24-hour specimen or > 0.3 g/l of protein in urine, or two positive dipstick readings in the absence of a urinary tract infection.

In a previously described custom-made targeted exomic sequencing protocol, we combined Illumina sequencing libraries for capture and sequencing with Nimblegen sequence capture (23, 24). The subjects and methods of this study have been described in detail previously (19, 21, 24, 25).

### Statistics

Sequence data were analyzed in PLINK/Seq, Plink (26)and R programs. Quality control before meta-analysis included removal of singleton and monomorphic variants, removal of sites with > 10% missing data in the targeted sequencing or a significant departure from Hardy-Weinberg equilibrium in controls (p < 0.001). Analyses of the significant associations were performed by the Fisher’s exact test. P-values < 0.05 were considered to indicate a significant difference. The tail of the p-value distribution of benign variants was as expected, suggesting that the overall study design and quality control were successful without multiple test adjustments. In addition to an appropriate statistical probability test, odds ratios (OR) with 95% confidence intervals (CI95) were calculated for all variants.

### Study approval

All subjects provided a written informed consent for the study. The FINNPEC study protocol was approved by the coordinating Ethics Committee of the Hospital District of Helsinki and Uusimaa (permit number 149/E0/07). National FINRISK study description and ethical approvals are available online: https://www.thl./documents/10531/1921702/2015+FINRISK+description_for_researchers_nal.pdf/fc952cba-86f6-4ef5-8ef2-fa13c23173c3.

## Results

To analyze if variants in genes coding for 40 components of the complement system are associated with PE, we performed association testing on Finnish preeclamptic mothers (609) and controls (2,092). The studied genes are listed in Supplementary table 1 and results of these genetic association analyses are listed in Table 1, Of the 15 associating variants, 10 were rare (MAF < 0.1) and, of these, 7 were missense variants. The lack of inflated p-values in comparison of benign and putatively functional variants indicates that confounders such as stratification are not causing false positives.

**Figure 1:**
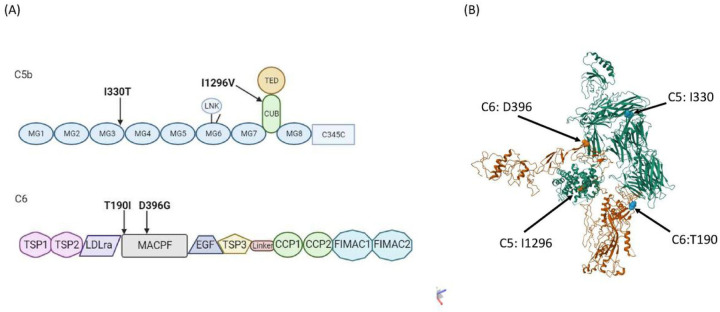
(A) The domain structure of C5b (top) and C6 (bottom) with PE associating variants indicated. The C5 variants are located in exon 31/41 (I330) coding the Immunoglobulin-like domain 3 (MG3) and in exon 9/41 (I1296) coding the conserved complement-activating CUB (complement C1r/C1s, Uegf, Bmp1) domain Both C6 variants in exons 6/16 (T190) and 12/16 (D396) are located in the membrane attack complex/perforin domain (MACPF). (B) The protein structure of C6 (orange) in complex with C5b (green) with the PE-associating amino acids’ positions indicated. The D396 is located directly in the interaction site between the two molecules. The structural model is based on construct 4A5W (37).

Interestingly, two rare missense variants in both *C5* and *C6* were found to associate with PE (Fig. 1A). Of these, the rs200674959 (I1296V), located in conserved complement-activating CUB (complement C1r/C1s, Uegf, Bmp1) domain and rs147430470 (I330T) located in Macroglobulin-like domain 3 (MG3) in *C5*, were predisposing variants (OR = 24.13, CI95 = 1.25–467.43; p = 0.01 and OR = 22.75, CI95 = 1.17–440.78; p = 0.01, respectively). Also, the rs41271067 (D396G) variant in *C6* was predisposing (OR = 2.93, CI95 = 1.18–7.10; p = 0.01), while rs114609505 (T190I) had a protective effect (OR = 0.47, CI95 = 0.22–0.92; p = 0.02). Both of the associating *C6* variants were located in the membrane attack complex/perforin domain (MACPF). No associating variants were discovered in the MAC inhibitor protectin *(CD59)*.

Other potential associations were found in rare predisposing variants in genes coding for CRP (rs1800947, p = 0.02), CFI (rs200040240, p = 0.003), C3 (rs45532534, p = 0.011) and C5 (rs200674959, p = 0.011, discussed above). The strongest protective associations were found in common variants in genes coding for CFHR4 (rs7417769, p = 0.012) and C8B (rs605648, p = 0.01). To summarize, associating variants were found in genes coding for components of the classical and alternative pathways, but not in the activating components of the lectin pathway. Importantly, six variants were found in the common terminal pathway.

## Discussion

In this study we identified that rare missense variants in genes coding for C5 and C6 of the terminal pathway of complement system activation are associated with PE. We have previously described genetic associations of PE to C3, complement receptors CR3 and CR4 and to the key regulator factor H. Taken together our findings suggest that abnormalities in the alternative and terminal pathway are of particular importance to PE pathogenesis. Potential or known disease associations of the PE-associated variants discovered in this study are scarce in literature.

The terminal pathway of complement activation has received less attention in PE studies than the classical or alternative pathways. CD59 is the only membrane-bound regulator of the terminal pathway, which is attached to the surface of the endothelium and trophoblast cells by a glycophosphatidylinositol (GPI) anchor. The plasma levels of CD59 are increased in PE and associated with end-organ injury related laboratory measures often observed in severe PE, such as elevated liver enzymes and lactase dehydrogenase and decreased platelet count (27). We have previously shown that CD59 is abundant in the placental trophoblasts, where it is exposed to damage by turbulent intervillous maternal circulation, a characteristic of the PE placenta caused by inadequate uterine arterial remodulation associated with the disease etiology (18, 28). It is possible that shear damage caused to the syncytiotrophoblast releases CD59 to maternal circulation thereby leaving the villous tissue vulnerable to aberrant complement attack. Terminal complement complex deposits have been localized in the fibrinoid material of the decidua of the basal plate, in the stroma of the chorionic villi and in the vessel walls, and their levels are increased in preeclamptic placentas (29). This may be exacerbated in patients with MAC-coding genetic variants. Both associating variants in *C6* are located in the membrane attack complex/perforin domain, which is responsible the MAC function of C6. The D396G is directly in the interaction site between C6 and C5b (Fig. 1B).

We have previously shown that genetic variants of complement component 3 (C3) are associated with susceptibility to PE (25). The protective variant rs2230201 in PE is known to relate to levels of C3 in serum as well as to associate with dense deposit disease (DDD) and systemic lupus erythematosus (SLE) (30–32). Furthermore, the rs2230201 observed in this study is in linkage disequilibrium (D’=1; Finnish population) with the rare variant rs190390034 in C3, which was previously found to be independently, and in a C3-haplotype, associated with increased risk for severe PE (25). Thus, the association of rs2230201 corroborates our previous finding.

CRP is an inflammation marker and trigger of the CP of complement activation. In addition, CRP controls the AP to promote iC3b-mediated clearance of debris by recruiting factor H (33). The minor PE-associated allele of rs1800947 in CRP has been found to be associated with a reduced plasma level of CRP in an elderly Finnish population (34). We found that the rs1800947 predisposes to PE (p-value = 0.021; OR 1.356 CI95 = 1.044–1.748). While the variant, also known as CRP2 is synonymous, it has been suggested to have a possible regulatory function. Studies in complex diseases have shown an association of rs1800947, for example, with systemic lupus erythematosus, the severity of prostate cancer and protection from cardiovascular disease (35–37).

This study adds to previous evidence by us and others indicating that in discordant patients, rare complement variants may play an important role in risk for PE. Variants of uncertain significance in genes coding for components of the terminal pathway of complement system may, in the context of physiological changes typical to PE become pathogenic. This is in support of the multiple hit theory of PE aetiology, where multiple factors, one being a rare complement variant, together create the perfect storm of disease-causing conditions.

## Figures and Tables

**Table 1:** Genetic variants within genes coding for components of the complement system that are associated with preeclampsia (p ≤ 0.05).

RSID	Gene name	P-value	OR (95% confidence interval)	MAF cases/MAF controls (%)	Consequence (distance from exon, base pairs)	ACMG/ClinVar	Role of gene
rs45532534	C3	0.01	13.80 (1.36 – 677.10)	0.33/0.02	intron variant (+39)	VUS/NA	AP activation
rs2230201	C3	0.04	1.17 (1.01 – 1.37)	30.61/26.08	synonymous variant R304=; TF binding site	Benign/Benign	AP activation
rs41258244	CD46	0.05	1.37 (0.995 – 1.863)	5.15/3.82	intron variant (−41)	VUS/Benign	CP and AP inhibition, surface-bound
rs1800947	CRP	0.02	1.36 (1.06 – 1.74)	7.66/5.69	synonymous variant, L184=	VUS/NA	CP activation, AP inhibition
rs3828032	CFHR2	0.020	0.825 (0.703 – 0.971)	26.93/22.22	intron variant (−20)	VUS/Benign	AP regulation
rs114727460	CFHR4	0.026	6.855 (0.981 – 75.743)	0.33/0.05	intron variant (−34)	VUS/NA	AP regulation
rs7417769	CFHR4	0.012	0.811 (0.688 – 0.956)	25.62/20.77	missense variant, N209S	VUS/Benign	AP regulation
rs35662416	CFHR5	0.033	1.819 (1.030 – 3.131)	1.81/1.00	missense variant, R359H	VUS/Likely-Benign	AP regulation
rs200674959	C5	0.01	24.13 (1.25 – 467.43)	0.25/0	missense variant, I1296V	VUS/VUS	TP activation
rs147430470	C5	0.01	22.75 (1.17 – 440.78)	0.25/0	missense variant, I330T	VUS/Likely-Benign	TP activation
rs41271067	C6	0.01	2.93 (1.18 – 7.10)	0.90/0.31	missense variant, D396G	VUS/Benign	TP activation
rs114609505	C6	0.02	0.47 (0.22 – 0.92)	0.82/1.73	missense variant, T190I	VUS/Likely-Benign	TP activation
rs605648	C8B	0.01	1.34 (1.08 – 1.66)	11.56/8.62	intron variant (−20)	VUS/Benign	TP activation
rs72670361	C8B	0.02	1.46 (1.056 – 2.007)	4.94/3.43	intron variant (+40)	VUS/NA	TP activation

RSID, SNP identifier; OR, odds ratio; MAF, minor allele frequency in total sample; VUS, variant of uncertain significance

NA, not available; C, complement; CP, classical pathway; TP, terminal pathway; AP, alternative pathway

## Data Availability

The datasets generated during and/or analysed during the current study are not publicly available due to patient confidentiality but are available from Professor Hannele Laivuori (hannele.laivuori@helsinki.fi) on reasonable request.
